# Allopregnanolone Improves Locomotor Activity and Arousal in the Aged CGG Knock-in Mouse Model of Fragile X-Associated Tremor/Ataxia Syndrome

**DOI:** 10.3389/fnins.2021.752973

**Published:** 2021-12-03

**Authors:** Jared J. Schwartzer, Dolores Garcia-Arocena, Amanda Jamal, Ali Izadi, Rob Willemsen, Robert F. Berman

**Affiliations:** ^1^Program in Neuroscience and Behavior, Department of Psychology and Education, Mount Holyoke College, South Hadley, MA, United States; ^2^The Jackson Laboratory, Clinical and Research Services, Sacramento, CA, United States; ^3^Department of Neurological Surgery, University of California, Davis, Davis, CA, United States; ^4^Department of Clinical Genetics, Erasmus MC, Rotterdam, Netherlands; ^5^M.I.N.D. Institute, University of California, Davis, Davis, CA, United States

**Keywords:** **:** FXTAS, allopregnanolone, mouse, behavior, adult neurogenesis

## Abstract

Carriers of the fragile X premutation (PM) can develop a variety of early neurological symptoms, including depression, anxiety and cognitive impairment as well as being at risk for developing the late-onset fragile X-associated tremor/ataxia syndrome (FXTAS). The absence of effective treatments for FXTAS underscores the importance of developing efficacious therapies to reduce the neurological symptoms in elderly PM carriers and FXTAS patients. A recent preliminary study reported that weekly infusions of Allopregnanolone (Allop) may improve deficits in executive function, learning and memory in FXTAS patients. Based on this study we examined whether Allop would improve neurological function in the aged CGG knock-in (CGG KI) dutch mouse, B6.129P2(Cg)-Fmr1^tm2Cgr^/Cgr, that models much of the symptomatology in PM carriers and FXTAS patients. Wild type and CGG KI mice received 10 weekly injections of Allop (10 mg/kg, s.c.), followed by a battery of behavioral tests of motor function, anxiety, and repetitive behavior, and 5-bromo-2′-deoxyuridine (BrdU) labeling to examine adult neurogenesis. The results provided evidence that Allop in CGG KI mice normalized motor performance and reduced thigmotaxis in the open field, normalized repetitive digging behavior in the marble burying test, but did not appear to increase adult neurogenesis in the hippocampus. Considered together, these results support further examination of Allop as a therapeutic strategy in patients with FXTAS.

## Introduction

Approximately 1.5 million individuals in the US are carriers of the Fragile X premutation (PM), with prevalence estimates ranging from 1:209 to 1:250 in females and 1:430 to 1:800 in males (Hunter et al., 2008; Hantash et al., 2011; Tassone et al., 2012). Many carriers develop neurological and psychological problems over their lifespan including anxiety, depression, and poor motor performance (Aziz et al., 2003; Moore et al., 2004; Cornish et al., 2005; Farzin et al., 2006; Coffey et al., 2008; Koldewyn et al., 2008; Bourgeois et al., 2011; Goodrich-Hunsaker et al., 2011). Carriers of PM are also at risk for developing Fragile X-associated tremor/ataxia syndrome (FXTAS), a late onset neurodegenerative disorder characterized by tremors, ataxia, brain atrophy and cognitive decline (Hagerman et al., 2001). The chances of developing FXTAS increase dramatically with age, with approximately 40% of males and 8–11% of female PM carriers over the age of 50 developing FXTAS (Rodriguez-Revenga et al., 2009). Indeed, FXTAS may be one of the more common causes of tremor/ataxia in older adults (Jacquemont et al., 2004). Because of the dramatic increase in the number of individuals reaching the age of 65, and increasing life expectancy, the numbers of FXTAS patients will increase accordingly, further highlighting the importance of research on PM and FXTAS (Jacquemont et al., 2004). At the present time there are no effective treatments to improve neurological function and overall quality of life for many affected PM carriers and FXTAS patients (Hagerman et al., 2008, 2009, 2010; Berry-Kravis et al., 2011). Therefore, it is extremely important to understand the underlying pathology in FXTAS, establish its developmental time course, and develop rational treatment strategies.

The goal of this preclinical study was to determine whether chronic treatment with the neurosteroid Allopregnanolone (Allop, 3α-hydroxy-5α-pregnan-20-one) would improve motor function and anxiety-like behaviors in a knock-in mouse model (i.e., CGG_ex_ KI) of the PM and FXTAS (Willemsen et al., 2003; Van Dam et al., 2005; Berman and Willemsen, 2009). This mouse KI model recapitulates much of the pathology in the PM and FXTAS (Berman and Willemsen, 2009). This includes a 2–3-fold elevation in *Fmr1* mRNA in brain and the accumulation over time of ubiquitin-positive intranuclear inclusions in both neurons and astrocytes. The KI model also exhibits motor deficits on the rotarod and ladder walking tasks (Van Dam et al., 2005; Hunsaker et al., 2011), cognitive impairments in several spatial memory tasks (Van Dam et al., 2005; Hunsaker et al., 2009), and increased anxiety in the elevated plus-maze (Berman and Willemsen, 2009), modeling clinical symptoms often seen in PM carriers and in patients with FXTAS.

Allop is a neurosteroid that enhances GABA_A_ function, stimulates neurogenesis and has been shown to improve cognitive function in animal models of neurodegenerative disease including Alzheimer’s disease (Brinton and Wang, 2006a,b; Wang et al., 2010). Allop has also has been found to enhance adult neurogenesis in the hippocampal subgranular zone (SGZ) (Genazzani et al., 1998; Kim et al., 2006; Herd et al., 2007; Nilsen et al., 2007; Wang et al., 2010; Chen et al., 2011; Noorbakhsh et al., 2011; Turkmen et al., 2011; Holden et al., 2012; Singh et al., 2012; Sun et al., 2012). Allop appears to stimulate neurogenesis in the hippocampus through a GABA_A_ mediated increase in intracellular calcium, leading to increased expression of genes for proteins that promote cell proliferation (Wang et al., 2005, 2008). This is relevant because GABAergic transmission appears to be abnormal in CGG_ex_ KI mice. D’Hulst et al. (2009) reported that GABA_A_ receptor subunits, transporters (GAT 1 and 2), and GAD are upregulated in the cerebellum of CGG_ex_ KI mice, suggesting a compensatory response to decreased GABAergic transmission. Levels of the GABA vesicular transporter VGAT are also lower in hippocampal neurons cultured from CGG_ex_ KI mice suggesting reduced presynaptic levels of GABA (Cao et al., 2012). In addition, reduced GABAergic signaling in cultured neurons from CGG_ex_ KI mice results in burst-firing of neurons and abnormal patterns of Ca^+2^ oscillations that can be rescued by culturing neurons in the presence of Allop (Cao et al., 2012). Therefore, in this study we examined the ability of Allop treatment to ameliorate behavioral pathology in a mouse model of the PM and FXTAS.

A recent open-label study assessed whether Allop given as 12 weekly infusions (2–6 mg) to 6 FXTAS patients would improve clinical symptoms, brain electrophysiological activity, and MRI measurements of brain deterioration and white matter pathology (Wang et al., 2017). Treatment improved executive functioning, episodic memory and learning, and increased N400 repetition effect amplitude as an index of brain activity. Overall deterioration of the brain on MRI was not significantly affected, although some patients showed improvement. In a separate report in 2019, plasma pharmacometabolomics and lymphocytic mitochondria function were assessed at baseline and within 48 h from the last infusion, and Allop treatment altered GABA metabolism and reduced markers of oxidative stress (Napoli et al., 2019). Considered together, results of this limited pilot clinical research project indicate that Allop may improve psychological and cognitive performance in FXTAS, and that further research is warranted, including preclinical studies such as the present study.

## Materials and Methods

### Animals

**FIGURE 1:**
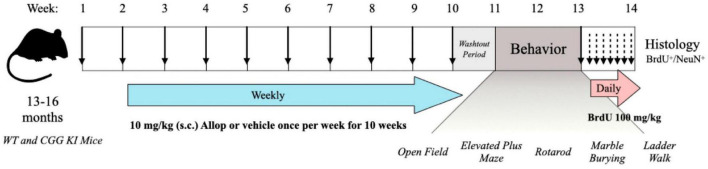
Timeline for the experimental procedures. WT and CCG KI mice between the ages of 13–16 months old were treated once weekly with allopregnanolone (Allop) (10 mg/kg s.c.) or β-cyclodextrin vehicle control. Following the last injection, mice were left undisturbed for a 1-week drug washout period and then assessed for behavioral differences across a battery of motor and arousal tasks. Then, mice were injected once daily with 5-bromo-2′-deoxyuridine (BrdU, 100 mg/kg i.p.) for seven consecutive days and their brains were then processed for evidence of neurogenesis.

Twenty-three male B6.129P2(Cg)-Fmr1^tm2Cgr^ “dutch CGG KI mice” (CGG KI) aged 13–16 months old, and 18 aged-matched wild type (WT) littermates were derived from a total of 18 litters bred at the UC Davis Neurotherapeutics Institute. The range of CGG trinucleotide repeat expansions for CGG KI mice was 67–197 (Mean 112 ± 10.5), and 10.0 ± 0.4 for WT mice. The mice were randomly assigned to receive 10 once-weekly injections of 10 mg/kg, s.c. Allopregnanalone (Allop) or β-cyclodextrin vehicle control (Veh) in one of 4 treatment groups: wild type-vehicle (WT-Veh; *n* = 9), wild type-Allop (WT-Allop; *n* = 9), CGG KI-vehicle (CGG KI-Veh; *n* = 11), CGG KI-Allop (CGG KI-Allop; *n* = 12). This drug injection protocol was patterned after that reported to improve cognitive performance in a mouse model of Alzheimer’s disease (Singh et al., 2012), and the Allop treatment paradigm was designed to avoid the potential cognitive impairments observed with acute exposure (Johansson et al., 2002, 2016). In order to avoid any transient effects of Allop as an acute GABA_A_ receptor modulator, mice were given a 1-week washout period after the last Allop injection before being evaluated across a series of behavioral tasks to assess motor coordination, balance, locomotor activity, and arousal as outlined in Figure 1. All experiments were conducted during the light phase of the light/dark cycle and were pre-approved by the University of California, Davis IACUC and in accordance with the policies of the National Institutes of Health for the humane care and use of laboratory animals.

### Genotyping

CGG repeat length was determined using methods previously described by Hunsaker et al. (2011). Briefly, DNA was extracted from mouse tails by incubation with 10 mg/mL Proteinase K (Roche Diagnostics; Mannheim, Germany) in 300 μL lysis buffer containing 50 mM Tris-HCl, pH 7.5, 10 mM EDTA, 150 mM NaCl, 1% SDS overnight at 55°C. One hundred μL saturated NaCl was then added and the suspension was centrifuged. One volume of 100% ethanol was added, gently mixed, and the DNA was pelleted by centrifugation and the supernatant discarded. The DNA was washed and centrifuged in 500 μL 70% ethanol and then dissolved in 100 μL milliQ-H_2_O. CGG repeat lengths were determined by PCR using the Expanded High Fidelity Plus PCR System (Roche Diagnostics). Approximately 500–700 ng of DNA was added to 50 μL of PCR mixture containing 2.0 μM/L of each primer, 250 μM/L of each dNTP (Invitrogen; Tigard, OR), 2% dimethyl sulfoxide (Sigma-Aldrich; St. Louis, MO), 2.5 M Betaine (Sigma-Aldrich), 5 U Expand HF buffer with Mg (7.5 μM/L). The forward primer was 5′-GCTCAGCTCCGTTTCGGTTTCACTTCCGGT-3′ and the reverse primer was 5′-AGCCCCGCACTTCCACCACCA GCTCCTCCA-3′. PCR steps were 10 min denaturation at 95°C, followed by 34 cycles of 1 min denaturation at 95°C, annealing for 1 min at 65°C, and elongation for 5 min at 75°C to end each cycle. PCR ended with a final elongation step of 10 min at 75°C. DNA CGG band sizes were determined by running DNA samples on a 2.5% agarose gel and staining DNA with ethidium bromide.

### Open Field

Locomotor behaviors were tested by placing mice in an empty Plexiglas arena (30 cm × 30 cm × 38 cm) and allowing them to freely explore the environment for 5 min. The total time spent in the center and margin of the arena as well as the number of line crosses in a 3 × 3 grid were counted by researchers blind to genotype and treatment conditions.

### Marble Burying

Mice were habituated for 10 min to clean Plexiglas cages (37 cm × 14 cm × 12.5 cm) filled with a 4 cm thick layer of clean corncob bedding for 10 min. Following habituation, animals were returned to their home cage and 15 glass marbles were laid out in five rows of three marbles placed equidistance apart. Mice were then returned to the cages and allowed to explore under dim illumination for 10 min. At the end of the 10 min period, animals were gently removed from the testing cages and the number of marbles buried was recorded. Only marbles covered by 75% or more bedding were counted as buried.

### Rotarod

Balance and motor coordination were assessed using a Rotamex-5 rotarod with infrared photocell detection (Columbus Instr., Columbus, OH). Mice were first given an initial training session by placing them on the rotarod rotating at a constant speed of 4 RPM for 120 sec. Mice that fell were immediately reloaded and allowed to complete the training session. The following day, mice were placed on the rotarod at an initial speed of 4 RPM that accelerated by 1.0 RPM every 10 s. A trial was terminated when a mouse fell from the rod at which time the latency to fall was recorded. Each mouse was tested twice. Mean performance time was defined as the average time the mouse remained on the rotarod across trials.

### Elevated Plus Maze

Anxiety in the mice was assessed using the elevated plus maze (Handley and Mithani, 1984; Walf and Frye, 2007). The Plexiglas apparatus consisted of two open arms (30 cm × 5 cm × 0.5 cm) and two perpendicular closed arms (30 cm × 5 cm × 15 cm) extending from a central platform. The entire maze was elevated approximately 1 m from the floor. Mice were placed in the central platform and allowed to freely explore the maze for 5 min. The total number of entries in the open arm, as defined by all four paws outside the central zone, as well as the total time spent in the open and closed arms were recorded. Percent of open arm exploration time was calculated as the time in the open arm divided by the total time in both the open and closed arms.

### Ladder Walk

The ladder walk task was conducted as previously described (Hunsaker et al., 2011). The apparatus consisted of two, 28 cm tall × 65 cm long black walls separated by 10 cm. The floor was elevated 10 cm from the bottom of the walls and was made from 43 parallel 1 mm diameter bars separated by 1.5 cm. Mice were placed in the apparatus and allowed to freely explore the apparatus for 2 min while being video recorded. The digital recordings were scored for the number of times a foot (forelimb or hindlimb) slipped through the floor bars as previously described (Hunsaker et al., 2011). The digital recordings were scored independently by two experimenters blinded to the genotype and treatment of the animals.

### Histology

Immediately following the final behavioral task, mice were injected once daily with 5-bromo-2′-deoxyuridine (BrdU) (100 mg/kg, i.p.) for seven consecutive days and then sacrificed to quantify hippocampal neurogenesis as previously described (Wojtowicz and Kee, 2006). Briefly, brain tissue was fixed with 4% paraformaldehyde, flash frozen, and serial 40 μm sections were cut anterior-posterior through the hippocampus. Sections were mounted on glass slides, rinsed 3 times for 10 min each with 0.1 M phosphate buffered saline (PBS) followed by 0.5% hydrogen peroxide in methanol for 15 min. Following another series of rinses, sections were submerged in 2N HCL solution for 20 min at 37 degrees Celsius, rinsed 3 times for 10 min each, and placed in blocking solution of 3% BSA, 3% goat serum, and 0.3% triton X for 1 h. Sections were then incubated for 18 h in blocking solution containing 1:50 anti-BrdU antibody (Accurate Chemical) and 1:100 anti-NeuN (EMD Millipore). The following day, sections were rinsed 6 times for 5 min each with 0.1 M PBS containing 0.3% Triton X and then incubated in blocking solution containing fluorescent-labeled secondary antibodies for 2 h. Sections were counter stained in a 1:500 solution of DAPI, rinsed 6 times for 5 min. each in 0.1 M PBS, and coverslipped with fluoromount G. The number of BrdU^+^ and double labeled BrdU^+^/NeuN^+^ cells were quantified in the subgranular zone of the dentate gyrus, defined as a narrow band 1–2 cells thick between the granule cell layer and the hilus (Kempermann et al., 1997), on every fifth section. Co-localization was determined by focusing through each section and toggling between filter sets to visually establish that immunofluorescence for BrdU and NeuN was from the same cell.

### Statistical Analysis

Data were analyzed using R version 4.0.4 (2021) using the “stats” and “pscl” packages. Mortality measures (i.e., number of deceased mice throughout the study) were evaluated across genotype and treatment conditions using chi-square test for independence. For behavioral measures, assumptions of normality and linearity were evaluated using the Shapiro-Wilks test and assessed visually using qqplots and histograms. Count data (number of open arm entries, marble burying, and ladder walk slips, and cell counts) containing excess numbers of zeros were assessed using Zero-Inflated Poisson regression followed by *post hoc* planned comparisons. Similarly for continuous measures (open field, rotarod), data were analyzed using Wilcoxon-Mann-Whitney planned comparisons to determine if (1) wild type mice treated with vehicle (WT-Veh) differed significantly from vehicle-treated CGG KI mice (CGG KI-Veh); (2) CGG KI mice treated with Allop (CGG KI-Allop) differed significantly from CGG KI-Veh mice; and (3) if wild type mice treated with Allop (WT-Allop) differed significantly from WT-Veh. All analyses were two-tailed, and values of *p* ≤ 0.05 were considered statistically significant. All graphs show sample medians and inter-quartile range (IQR).

## Results

### Mortality and Body Weight

A total of 4 CGG KI mice and 2 wild type mice died during the time-course of the study. Of those deceased, 1 CGG KI vehicle-injected and 2 wild type Allop-injected mice were euthanized before the end of the injection series due to skin lesions at the site of the subcutaneous injections (Table 1). Chi-square analysis confirmed that there were no differences in the number of mice that died or were euthanized between genotype [*X*^2^ (1, *N* = 41) = 0.81, *p* = 0.37] or drug treatment, [*X*^2^ (1, *N* = 41) = 0.04, *p* = 0.84]. There were no significant differences in body weight among groups before or after the last injection of either saline or Allop, and no significant change in body weight between the 1st and 10th injection due to treatment or genotype.

**TABLE 1:** Sample sizes for each genotype and treatment group.

Genotype	Treatment	Alive	Deceased
Wild type	Vehicle	9	0
Wild type	Allop	7	2
CGG repeat	Vehicle	9	2
CGG repeat	Allop	10	2

*There were no significant differences in the number of mice that died or were euthanized between groups.*

### Locomotor Activity and Arousal

**FIGURE 2:**
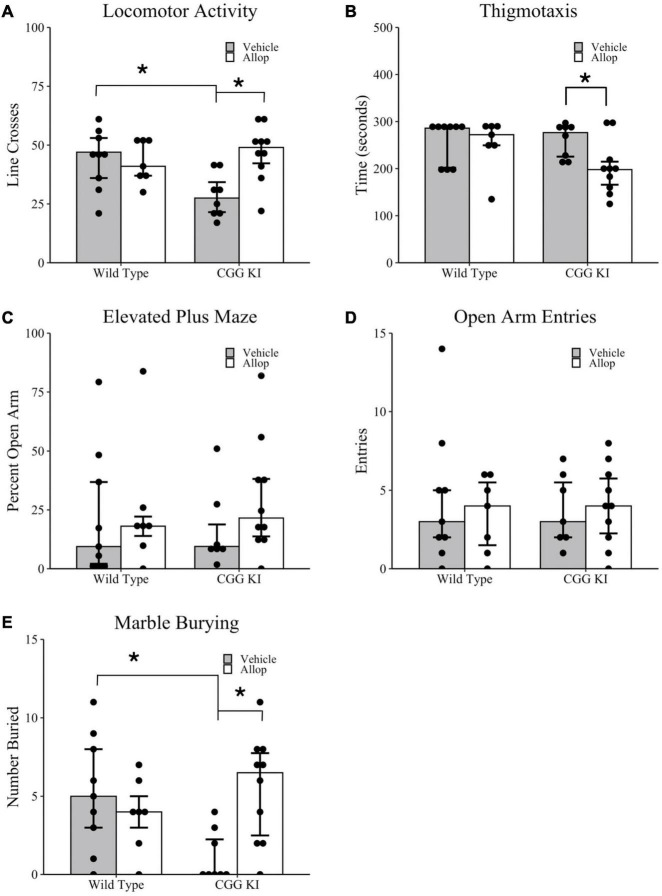
Locomotor activity, arousal, and anxiety-associated behaviors in the CGG KI and wild type mice. Mice were treated for 10 weeks with Allop or vehicle control and evaluated for locomotor activity and thigmotaxis in the open field task **(A,B)**, anxiety-like measures in the elevated plus maze **(C,D)**, and repetitive digging behavior in the marble burying task **(E)**. Reported are **(A)** the number of lines crossed during a 5-min open field exploration; **(B)** the total time spent exploring the margins of the open field arena (i.e., thigmotaxis); **(C)** the percent time exploring the open arms in the elevated plus maze, as well as **(D)** the total number of entries into the open arms; **(E)** the percent of marbles buried in the marble burying task. *p < 0.05 as determined by Wilcoxon-Mann-Whitney (Locomotor Activity and Thigmotaxis) or Zero-Inflated Poisson Regression (Elevated Plus Maze and Marble Burying) followed by pairwise planned comparisons. Plots represent individual mice; bars represent median ± IQR. Wild type-vehicle (*n* = 9), wild type-Allop (*n* = 7), CGG KI-vehicle (*n* = 8), CGG KI-Allop (*n* = 10).

Locomotor and anxiety-related behaviors for CGG KI and wild type mice were measured in the open field and elevated plus maze tasks (Figures 2A–D). Overall activity in the open field was quantified by the number of line crosses over a 5 min period. As shown in Figure 2A, CGG KI-Veh mice had significantly fewer line crosses (i.e., lower activity) compared to WT-Veh mice, and this effect was ameliorated in the CGG KI mice by Allop treatment. Statistical analysis of locomotor activity in the open field, measured by the number of line crosses, confirmed that activity was significantly lower in CGG-Veh mice compared to WT-Veh mice (*p* = 0.018). Allop treatment did not significantly affect Wild type mice (i.e., WT-Veh vs. WT-Allop, *p* = 0.790), but significantly increased activity in the CGG KI mice (CGG KI-Veh vs. CGG KI-Allop, *p* = 0.007). In fact, Allop treatment restored locomotor activity in the CGG KI mouse to a level similar to vehicle-treated wild type mice.

As shown in Figure 2B, there was a significant effect of Allop treatment on time spent in the margin for CGG KI mice. Planned comparisons showed that WT-Veh mice did not differ significantly in margin time from either WT-Allop (*p* = 0.831) or CGG KI-Veh (*p* = 0.699). However, CGG KI-Allop treated mice spent less time in the margin compared to CGG KI-Veh (*p* = 0.050).

In the elevated plus maze, there was no difference in percent time in the open arms between vehicle treated mice of both genotypes (*p* = 0.918) and no effect of treatment on CGG KI-Allop (*p* = 0.133) or WT-Veh (*p* = 0.491). On average, mice only spent approximately 24% of their exploration time in the open arms of the maze (Figure 2C). Importantly, there were no differences in the total number of open arm entries between genotypes (z = 0.04, *p* = 0.631) or treatment (z = 0.02, *p* = 0.851), suggesting that motor activity did not impact elevated plus maze performance (Figure 2D).

Figure 2E shows the results of Allop treatment on marble burying used as a test of repetitive digging behavior (Thomas et al., 2009). There was a significant treatment by genotype interaction (*z* = 2.26, *p* = 0.024), in the number of marbles buried in a 10-min marble burying task. Planned comparisons confirmed that CGG KI-Veh mice buried fewer marbles than WT-Veh and CGG KI-Allop mice (*p* < 0.001 for both comparisons).

### Motor Coordination

**FIGURE 3:**
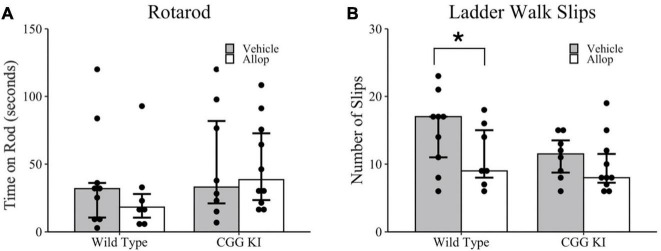
Behavioral assessments of balance and motor coordination in response to Allop in the wild type and CGG KI mouse. Following 10 weeks of Allop treatment mice were evaluated for changes in balance on the rotarod task and motor coordination in the ladder rung task. **(A)** No differences were observed in the average time to fall in the rotarod task. **(B)** In the ladder rung task, mice were evaluated for total number of slips. *p < 0.05 as deteremined by Wilcoxon-Mann-Whitney (Rotarod) or Poisson Regression (Ladder Walk) followed by pairwise planned comparisons. Plots represent individual mice; bars represent median ± IQR. Wild type-vehicle (*n* = 9), wild type-Allop (*n* = 7), CGG KI-vehicle (*n* = 8), CGG KI-Allop (*n* = 10).

Motor coordination was evaluated in the rotarod (Figure 3A) and ladder walk tasks (Figure 3B). In the rotarod task, there were no differences in total time on the rotarod between genotypes, WT-Veh compared to CGG KI-Veh (*p* = 0.630), and no differences between treatment conditions, CGG KI-Veh compared to CGG KI-Allop (*p* = 0.897) and WT-Veh compared to WT-Allop (*p* = 0.536). On the ladder walk test, a poisson regression revealed a marginally significant effect of treatment (*z* = 1.93, *p* = 0.053) based on genotype (*z* = 2.08, *p* = 0.038). Specifically, Allop treatment in WT mice reduced the number of foot slips compared to WT-Veh controls (*p* = 0.051), but no differences were observed between Allop and Vehicle treatment in CGG KI mice (*p* = 0.425) (Figure 3B).

### Dentate Gyrus Neurogenesis

**FIGURE 4:**
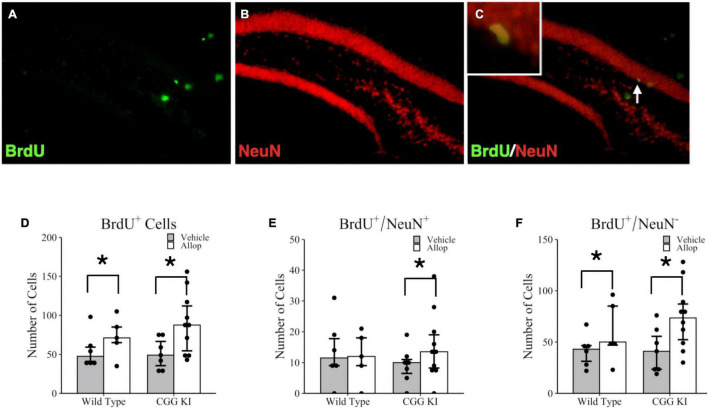
Hippocampal cell proliferation and adult neurogenesis in the brains of CGG KI and wild type mice. Fluorescent photomicrographs of **(A)** BrdU^+^ cells (green fluorescence) in the subgranular zone of the hippocampus. **(B)** Neurons were labeled with NeuN (red fluorescence), and **(C)** newly generated neurons are co-labled BrdU^+^/NeuN^+^ and appear yellow (high magnification insert). Images shown at 20x magnification. **(D)** The total number of BrdU^+^ cells in the subgranular zone of WT and CGG KI mice treated with Allop or vehicle for 10 weeks. **(E)** The total number of double labeled BrdU^+^/NeuN^+^ cells reflecting adult neurogenesis in the dentate gyrus of the hippocampus. **(F)** The number of new cells not labeled with NeuN (BrdU^+^/NeuN^–^) was calculated as the total number of number of BrdU^+^ cells in **(D)** minus the total number of BrdU^+^/NeuN^+^ in **(E)**. *p < 0.05 as determined by poisson regression (BrdU^+^ and BrdU^+^/NeuN^–^) or zero-inflated poisson regression (double-labeled BrdU^+^/NeuN^+^) analysis followed by pairwise planned comparisons. Plots represent aggregated count data of individual mice; bars represent median + IQR. Wild type-vehicle (*n* = 5), wild type-Allop (*n* = 6), CGG KI-vehicle (*n* = 7), CGG KI-Allop (*n* = 10). BrdU, 5-bromo-2′-deoxyuridine.

The effects of 10 weeks of once weekly Allop treatment on cell proliferation and neurogenesis in the hippocampus are shown in Figure 4. Images of the dentate gyrus labeled with BrdU (green fluorescence, Figure 4A) and NeuN (red fluorescence, Figure 4B) were quantified for the number of colocalized BrdU^+^/NeuN^+^ cells (yellow fluorescent cells; see insert, Figure 4C). Poisson regression analysis for the total number of BrdU^+^ cells confirmed a significant treatment effect of Allop (*z* = −8.42, *p* < 0.001) but not for genotype (*z* = −1.42, *p* = 0.157). Figure 4D shows a greater number of BrdU^+^ cells in both WT-Allop (*p* = 0.003) and CGG KI-Allop (*p* < 0.001) compared to genotype-matched vehicle controls. There was no significant effect of genotype on the total number of BrdU^+^/NeuN^+^ double-labeled cells in the dentate gyrus (Figure 4E), classified as adult proliferating neurons (i.e., adult neurogenesis), and the effect of AlloP treatment on BrdU^+^/NeuN^+^ double-labeled cells did not reach statistical significance (*z* = −1.67, *p* = 0.095). However, there was a significant treatment by genotype interaction (*z* = 2.46, *p* = 0.014). Planned comparisons confirmed a small increase in the number of double-labeled BrdU^+^/NeuN^+^ cells in CGG KI-Allop mice compared to genotype-matched CGG KI-Veh mice (*p* = 0.024). Figure 4F shows the total number of cells that labeled for BrdU, but not NeuN (BrdU^+^/NeuN^–^), calculated as the difference between total BrdU^+^ cells labeled and BrdU^+^/NeuN^+^ double labeled cells. This population of BrdU labeled cells likely includes proliferating astrocytes, oligodendrocytes and microglia, as well as young neurons not expressing NeuN although they were not further classified in this study. Interestingly, there was a significant effect of treatment on the number of non-neuronal BrdU^+^/NeuN^–^ cells (*z* = −8.57, *p* < 0.001). Planned group comparisons showed the number of BrdU^+^/NeuN^–^ cells was significantly larger in the CGG KI-Allop mice compared to the CGG KI-Veh mice (*p* < 0.001), and in the WT-Allop mice compared to genotype-matched WT-Veh controls (*p* < 0.001). No effect of genotype were observed between WT-Veh mice and CGG KI-Veh groups (*p* = 0.923).

## Discussion

The CGG KI mouse is a well-validated and established pre-clinical tool for modeling the hallmark behavioral and neurobiological features of the Fragile X PM and FXTAS. This includes motor dysfunction, ubiquitin-positive inclusions, and disruptions in GABAergic signaling (Brouwer et al., 2008; D’Hulst et al., 2009; Berman et al., 2014). While treatments for FXTAS are, to date, only palliative, it has been hypothesized that pharmacological agents that target the GABAergic system may improve neurological function or delay disease progression. In particular, Allop has undergone an initial open-label clinical trial to test its efficacy in reversing the motor and cognitive deficits in a small population of elderly men with FXTAS (Wang et al., 2017; Napoli et al., 2019). Given the high degree of construct validity observed in the CGG KI mouse, we tested whether a similar regimen of Allop in aged CGG KI mice would show efficacy in reducing the behavioral pathologies observed in this mutant mouse model. Our results demonstrated significant improvements in locomotor activity in the open field, but without concomitant improvements in coordination and balance in the rotarod or ladder walk tests. Together, these data provide additional evidence supporting a potential therapeutic effect of Allop on specific FXTAS-associated behavioral deficits.

In humans, premutation carriers show longer, slower motor movements and reaction times that worsen with age (Shickman et al., 2018). Our aged CGG KI mice showed similar deficits in locomotor activity and arousal given the observed reductions in line crosses in the open field task and reduced marble burying activity. The marble burying task in particular revealed a near absence of buried marbles in the CGG KI-Veh mice, likely due to deficits in motor activity and general arousal as a result of the insertion of the CGG-repeat. Treatment with Allop for 10 weeks restored these deficits in motor activity and arousal in both the marble burying and open field tasks to levels observed in wild type controls. These convergent phenotypes across multiple behavioral assays (i.e., open field and marble burying) serve as a measure of reliability in Allop’s restorative effects on motor activity. Interestingly, 10 weeks of Allop treatment did not further enhance arousal levels in wild type mice, underscoring the specificity of targeting GABAergic systems to restore, but not enhance, locomotor activity. While these improvements in motor activity would suggest a therapeutic potential for Allop, similar restorative responses were not observed in measures of anxiety, namely the elevated plus maze and thigmotaxis (time in center) measures in the open field arena. Performance on the elevated plus maze task declines with age in the C57BL/6 mouse strain, with aged-mice exhibiting fewer entries and reduced time in open-arm exploration (Sukoff Rizzo et al., 2018; Shoji and Miyakawa, 2019). Similar to our observations in the rotarod and ladder rung task, our observed similarities in elevated plus-maze performance between aged wild type and CGG KI mice would suggest that natural age-related declines in performance may be masking earlier anxiety-like effects of the CGG premutation reported in younger CGG KI mice. Therefore, the potential therapeutic effects of Allop treatment on anxiety-like measures remain inconclusive given our inability to effectively evaluate premutation-induced changes in elevated plus-maze performance.

Motor coordination, ataxia and balance are among the hallmark symptoms of FXTAS, and the CGG KI mouse is characterized to show similar deficits in movement and coordination including increases in foot slips in the ladder rung task (Hunsaker et al., 2011) and changes in rotarod performance (Van Dam et al., 2005). Unexpectedly, we did not observe these differences in motor function between our aged wild type and CGG KI mice in either test, regardless of Allop treatment. These results are similar to the study by Van Dam et al. (2005) who also found that rotarod performance did not differ significantly between 20-, 52-, and 72-week-old CGG KI and control mice. Only when genotypes were analyzed separately did they observe evidence of a significant age-related decline in rotarod performance in 52- and 72-week-old CGG mice compared to 20-week-old genotype-matched mice. While procedural differences between the present study and that of Van Dam et al. (2005) make it difficult to compare studies, such as rotarod apparatus (i.e., Rotamex 5 vs. Ugobasile), pretesting adaptation trials (i.e., a single vs. 2 min trials) and time between adaptation and testing (i.e., 24 h vs. immediate), it is clear that aged CGG KI and control mice did not differ significantly in either study. Previous studies have used a combination of young and old-aged mice (4–16 months) (Hunsaker et al., 2011; Wenzel et al., 2019), but the behavioral phenotype of CGG KI mice at advanced ages, such as the mice used in the present study, has not been well characterized. This is important given that C57BL/6 mice exhibit age-related changes in behaviors including increased anxiety and decreases in motor performance (Sukoff Rizzo et al., 2018; Shoji and Miyakawa, 2019). As a result, natural age-related declines in wild type mouse motor performance (e.g., rotarod and ladder rung test) may equal declines in the aged CGG KI mice, thereby masking differences between wild type and KI mice previously reported in younger mice. Moreover, Allop did not alter rotarod motor performance in either wild type or CGG KI groups, suggesting that weekly treatment with Allop does not improve motor function when started at an advanced age. In a recent pilot study of 6 individuals with FXTAS, 12-weeks Allop did not improve tremor or ataxia (Napoli et al., 2019) corroborating our preclinical findings in the CGG KI mouse. As a result, our data would suggest that targeting GABAergic function with weekly Allop treatment is not sufficient to restore motor coordination deficits resulting from age-related declines in motor function. However, it remains unexplored whether treatment at an earlier age could mitigate or delay the onset of pathology in premutation carriers before the full onset of FXTAS symptomatology.

Allop is an active metabolite of the naturally occurring neurosteroid progesterone with numerous actions in the central nervous system. Allop and other neurosteroids exert numerous classic and non-classic actions in the central nervous system, including promoting genomic and non-genomic cell-cycle functions modulating cell proliferation, cell shape, and gene expression (Garcia-Segura et al., 1996, 1999; Jordan, 1999; Nichols, 1999). Previous studies have demonstrated Allop’s ability to promote adult glial cell health and proliferation in addition to its effects on neurogenesis (for review see Faroni and Magnaghi, 2011). Allop is also an allosteric modifier of the GABA_A_ receptor and can stimulate neurogenesis in the hippocampus (Baulieu et al., 2001). Given the known proliferative effects of Allop on hippocampal neurogenesis both *in vitro* (Wang et al., 2005) and *in vivo* (Wang et al., 2010), we examined whether the locomotor and arousal improvements we observed in our CGG KI mice were accompanied by concomitant increases in adult neurogenesis in the dentate gyrus. Adult hippocampal neurogenesis has been demonstrated in all mammalian species studied to date including mouse (Kempermann et al., 1997; Zhao et al., 2006), rat (Kuhn et al., 1996), non-human primate (Gould et al., 1999) and human (Eriksson et al., 1998). The function of adult neurogenesis is still unclear, but a growing body of evidence indicates its importance in processing of sensory stimuli such as spatial cues necessary for behavioral discrimination (Tuncdemir et al., 2019). Ten weeks of treatment with Allop did not provide compelling evidence of increased neurogenesis in either aged wild type or CGG KI mice. However, this observation is tempered by the fact that in the present study group sizes were relatively small, and numbers of proliferating neurons in the dentate gyrus were also comparatively small, possibly due to the advanced age of the mice used in this study. Adult neurogenesis in the hippocampus declines with age in mice (Kempermann et al., 2002; Yang et al., 2015) and rats (Kuhn et al., 1996), although stable levels of human hippocampal adult neurogenesis have been reported throughout aging (Boldrini et al., 2018). Therefore, the potential for Allop to stimulate adult neurogenesis in the aged rodent hippocampus may be somewhat limited, making it more difficult to document potential drug effects on neurogenesis. It is also possible that the dosage or timing of injections were insufficient to stimulate neuronal proliferation in aged mice.

Ten weeks of Allop injections did significantly increase the total numbers of BrdU-labeled cells that were not NeuN^+^ in both wild type and CGG KI dentate gyrus. The identity of these proliferating cells was not further examined in the present study, and will need to be determined in future studies. One plausible explanation for this low level of BrdU^+^/NeuN^+^ double labeling may be a result of the timing of BrdU injections and time of sacrifice before histological evaluation. Specifically, Snyder et al. (2009) found that newly generated neurons do not express NeuN until 2–3-weeks after birth, a time period longer than used in the present study. Although we adopted a substantially different BrdU injection regimen to Snyder et al. (2009), their results open the possibility that many of the BrdU^+^ only cells in our AlloP-exposed mice may include immature neurons not yet expressing the NeuN neuronal marker (Snyder et al., 2009). If so, it is possible that the increase in BrdU^+^ cells (Figure 4D) included new neurons stimulated by AlloP treatment but not yet expressing NeuN. Alternatively, total number of BrdU^+^ cells in 4D includes astrocytes, oligodendrocyte precursor cells, oligodendrocytes and microglia. Gliogenesis in the adult mammalian hippocampus is well documented (Rietze et al., 2000; Wang et al., 2005; Nixon et al., 2008), and in a triple transgenic mouse model of Alzheimer’s disease (3xTgAD), chronic Allop treatment promoted not only neurogenesis, but oligodendrogenesis (Irwin and Brinton, 2014; Chen et al., 2020). It is notable that this increased proliferation of BrdU^+^ cells by Allop treatment was observed across wild type and CGG KI hippocampus, although planned comparisons indicated that the effect was mainly due to the drug’s effects in the CGG KI mouse hippocampus. Glia constitute roughly half of the cells of the central nervous system and provide important functions including homeostasis, neural development, migration, synaptogenesis, synaptic transmission and neural plasticity (Allen and Lyons, 2018). In view of the importance of glia for CNS function, and the possible occurrence of non-cell autonomous pathology from glial to neurons in FXTAS (Wenzel et al., 2019), it is possible that proliferation of one or more glial subtypes may have contributed to the behavioral results of the present study and this should be examined in future studies.

Considered together, our data provide evidence that 10 weeks of Allop treatment in aged-CGG KI mice improves deficits in locomotor activity and repetitive digging behavior in the marble burying test. Several behavioral measures evaluated in these aged mice, including rotarod and ladder walk, did not show differences between CGG KI and wild type controls in contrast to what has been reported in young CGG KI mice (Berman et al., 2014). This prevents the drawing of any conclusions about possible Allop effects on these types of behaviors. One possibility is that age-related declines in motor performance in very old wild type C57 mice may reach the same level as that of CGG KI mice, making it difficult to compare and identify impaired motor performance in aged CGG KI mice. Given the myriad effects of Allop in modulating both cellular and neurochemical systems in the central nervous system, it is important in the future to examine whether treatment with Allop in earlier stages of the PM pathology may be more effective in delaying the onset of the behavioral and histopathological deficits induced by the CGG repeat expansion.

## Data Availability Statement

The raw data supporting the conclusions of this article will be made available by the authors, without undue reservation.

## Ethics Statement

The animal study was reviewed and approved by the University of California, Davis, IACUC.

## Author Contributions

RW and RB contributed to conception and design of the study. JS, DG-A, AI, and AJ performed behavioral, genetic, and histological assays. JS and RB performed statistical analysis. JS and RB wrote the first draft of the manuscript. JS, DG-A, AJ, AI, RW, and RB contributed sections of the manuscript. All authors contributed to manuscript revision, read, and approved the submitted version.

## Conflict of Interest

The authors declare that the research was conducted in the absence of any commercial or financial relationships that could be construed as a potential conflict of interest.

## Publisher’s Note

All claims expressed in this article are solely those of the authors and do not necessarily represent those of their affiliated organizations, or those of the publisher, the editors and the reviewers. Any product that may be evaluated in this article, or claim that may be made by its manufacturer, is not guaranteed or endorsed by the publisher.
